# Prevention of the social isolation of older persons: the impact of community environmental satisfaction on social isolation

**DOI:** 10.3389/fpubh.2023.1177483

**Published:** 2023-06-05

**Authors:** Sen Ma, Gang Lou, Yifan Duan

**Affiliations:** ^1^College of Landscape Architecture and Arts, Northwest A&F University, Xianyang, China; ^2^College of Architecture, Chang’an University, Xi’an, China

**Keywords:** community, satisfaction with the environment, the older adult, optimal aging, social isolation

## Abstract

**Background:**

To explore how to prevent the social isolation of the older adult, this study constructed a model of the influence of community environmental satisfaction on the social isolation of the older adult from the three dimensions of environmental facilities, transportation, and supporting facilities around the community. Methods: The social network scale and environmental satisfaction scale were used to collect the sample data of nine communities in Xi’an, and the maximum likelihood estimation method was used to analyze the data and test the model.

**Results:**

(1) Environmental facilities, transportation, and community surrounding facilities promoted community environment satisfaction (*R*^2^ = 0.904). Among them, environmental facilities (*β* = 0.869) had the greatest impact on community environmental satisfaction, followed by transportation (*β* = 0.118), and surrounding facilities (*β* = 0.084) had the least impact on community environmental satisfaction. (2) Environmental satisfaction had a direct positive impact on social isolation. Among them, the impact of environmental satisfaction on friend isolation (*R*^2^ = 0.895, *β* = 0.829) was greater than that on family isolation (*R*^2^ = 0.718, *β* = 0.747).

**Conclusion:**

Environmental satisfaction can directly affect the social isolation of the older adult in the community and can be used as an intermediate variable of environmental facilities, transportation, and surrounding facilities in the community so that it can indirectly affect the social isolation of the older adult. The results of this study provide a scientific basis for the design of aging environments in the future.

## 1. Introduction

China’s aging problem is becoming increasingly serious. According to the latest census results of China, the number of people over 60 years old has reached 264.02 million, accounting for 18.70% of the total population (1). Research finds that most of the older adult in the community have lost their social participation and social interaction after retiring (2). American sociologist Burgess called this situation social isolation (3); that is, “people are passively or actively derailed from society, and their social interaction, interpersonal communication, activity participation and other communications are in isolation” (4). Studies on the consequences of social isolation and harm have conducted sufficient scientific research, but the research on preventing social isolation is limited (5). Therefore, the effect of community environment satisfaction on helping the older adult take the initiative to communicate, exercise, and travel is unknown. The prevention of social isolation requires further exploration.

### 1.1. Effects of social isolation

Social isolation has a serious impact on the quality of life of the older adult (6–8) and increases their sense of loneliness and produces a variety of negative emotions, thus causing a series of diseases, such as depression (9), stroke (10), and senile dementia (11). Long-term social isolation can even lead to extreme behaviors such as suicide (12). On the other hand, the health of the older adult directly influences their social isolation (13, 14), and a lack of exercise makes the older adult more prone to social isolation (15). Family factors can also produce social isolation in older people. Currently, the social isolation conditions of the older adult worldwide are not optimistic, and attention needs to be given to various social aspects.

### 1.2. Community environment satisfaction

The physical function of the older adult gradually declines with age, and the community and its surrounding space become the preferred place for the older adult to engage in activities and have social interactions. People are no longer limited to meeting through the daily use of the community environment but have begun to pursue other features. For example, Anthony Barnett et al. Claimed that the community environment and neighborhood relations has a great impact on the physical and mental health of the older adult (16). A good natural ecological environment in the community has a certain rehabilitation effect on the psychological and physiological health of the older adult (17). The evaluation of the quality of the community environment depends on people’s satisfaction with the community environment, among which community recreational facilities, healthcare facilities, and so on are important factors of community satisfaction (18).

Initial indicators were obtained through interviews with the target population and bibliometric analysis of CiteSpace. Combined with expert opinions, three indicators of environmental satisfaction were finally determined, namely, community environmental facilities, transportation, and supporting facilities around the community. On the basis of community satisfaction, the satisfaction index related to living was eliminated, and only the satisfaction index related to the outdoor activity space of community residents was targeted. Such indicators are more conducive to exploring the relationship between the community environment and social isolation.

### 1.3. Research hypothesis and model

**Figure 1:**
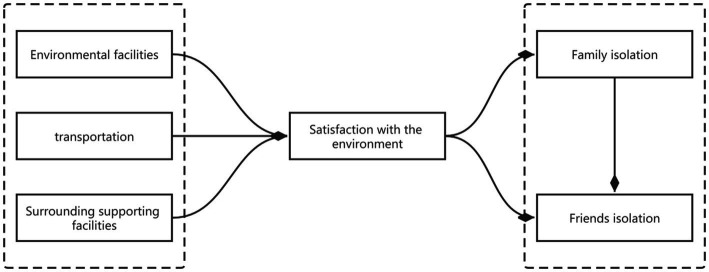
Impact model of environmental satisfaction and social isolation.

A structural equation model can solve many problems that cannot be directly observed and deal with the causal relationships between some fuzzy variables and multiple variables at the same time, making up for the shortcomings of traditional statistical methods (19). However, no study has used structural equation modeling to discuss the social isolation of the community environment and community older people. Based on this, this study identifies and analyzes the influencing factors of community environmental satisfaction and social isolation from the perspective of the community environment. In addition, a structural equation model, such as Figure 1, is constructed by taking environmental facilities, transportation, and supporting facilities as independent variables, environmental satisfaction as an intermediary variable, and family isolation and friend isolation as dependent variables. Based on the above discussion, the following hypotheses are proposed:

*H1. The facilities surrounding the community have a positive impact on the environmental satisfaction of the community.*

*H2. Community transportation has a positive impact on community environmental satisfaction.*

*H3. Community environmental facilities have a positive impact on community environmental satisfaction.*

*H4. Environmental satisfaction has a positive impact on family isolation.*

*H5. Environmental satisfaction positively impacts friend isolation.*

*H6. Family isolation has a positive impact on friend isolation.*

## 2. Materials and methods

### 2.1. The study area

The study was conducted in Xi’an, the capital of China’s Shaanxi province, with a total population of 12.6 million. According to the seventh census data, the older adult over 60 years old accounted for 16.02% of the city’s permanent population, an increase of 3.48 percentage points since 2010 (20). The population density of the older adult also shows that the aging speed is accelerating in Xi’an (21). This study selected three types of communities that contain mostly older adult residents from the three typical communities in the questionnaire (Table 1) (22). Among them, nine communities significantly differed in terms of the internal spatial structure, surrounding environment, and business environment. This allows a more comprehensive exploration of the problems related to the relationship between environmental and social isolation of older adult people in Xi’an to avoid data limitations.

**Table 1:** Community classification.

Community types	The name of the community
The business community	Dongshang community
	Laianyihui community
	Jiuzhangdeng community
Unit attached community	Jiaochang community
	Cheliang community
	Qianhu community
Street type old residential area	Yuze community
	Liangyi community
	Yangguang community

### 2.2. The research methods

The research questionnaire was divided into three parts according to the research objectives:

1. The general information scale measured gender, education level, age, marital status, monthly income, household registration, physical health status, etc.
2. The social isolation survey scale was the Lubben Social Network Scale, LSNS-6 (23) (a total of six items, three items for each dimension).
3. The environmental satisfaction scale contains 22 items, including 4 items on environmental satisfaction, with 8 questions in the environmental facilities dimension, 3 questions in the supporting facilities dimension, 3 questions in the transportation dimension, and 3 questions in the community service dimension.

The environmental satisfaction questionnaire and the social isolation questionnaire used a 7-point Likert scale (24, 25) (each item was assigned 1–7 points, with 7 points indicating “highly approve” and 1 point indicating “highly disapprove”; some items were trap questions, and such questions were revere scored).

### 2.3. Data collection

The survey period was from March to May 2022, and the participants were residents aged ≥60 years old, in good health, with autonomy and cognitive ability from the above nine communities. The questionnaire was administered during random interviews in the community. To ensure the accuracy of the survey data, the author explained the questionnaire to the participants individually to ensure that the participants fully understood the meaning of the questionnaire before completing it. To ensure the quality of the sample, the following method was used to screen the final questionnaire (26):

1. Some reverse-scored questions were added to the questionnaire, and if participants gave inconsistent answers to the positive and negative versions of a question, the questionnaire was considered invalid.
2. It took approximately 2–5 min to complete the questionnaire. The participants who completed the questionnaire within one minute were considered to have not answered the questionnaire seriously, and the questionnaire was regarded as invalid.

In the final field survey, a total of 220 questionnaires were distributed, and all 220 were recovered. Of these, 210 were valid, for a questionnaire recovery efficiency of 89%. Therefore, the amount of data in this study meets the requirements of the structural equation model (27–30).

### 2.4. Data processing

SPSS 26.0 software was used for statistical description and reliability testing of the data to determine the data reliability. Amos Graphics CLI software was used to construct a structural equation model to verify the variable relationship and test the model fitting degree. Thus, the hypothesized association between variables is verified to determine whether the hypothesis holds.

## 3. Results and analysis

### 3.1. Descriptive statistics

According to the analysis of the survey data (Table 2), 92 (43.8%) were male and 118 (56.2%) were female. Thus, the number of males and females was relatively balanced. In terms of academic qualifications, 89 of the participants (42.3%) had a junior high school education, 50 (23.8%) had a high school education, 31 (12.3%) had a secondary school education, and 26 (12.3%) had a junior college education or above. The number of older adult people with a high education level was low, and they primarily came from the commercial community and the old community attached to the unit.

**Table 2:** Basic information description.

Older population profile	
Age, M (SD)	75.33 (7.650)
Gender female, *n* (%)	118 (56.2%)
Record of formal schooling, *n* (%)	
Below junior high school	101 (48.1%)
Junior high school	50 (23.8%)
High school	33 (15.7%)
College or above	26 (12.3%)
Community, *n* (%)	
Dongshang community	24 (11.4%)
Laianyihui community	23 (11.4%)
Jiuzhangdeng community	22 (10.5%)
Jiaochang community	24 (11.4%)
Cheliang community	22 (10.5%)
Qianhu community	23 (11.0%)
Yuze community	24 (11.4%)
Liangyi community	22 (10.5%)
Yangguang community	26 (12.4%)
Position before retirement	
Cadre leader	25 (12%)
Worker	103 (49%)
Freelancer	82 (39%)

Second, for the daily travel mode of the older adult in the various communities, the study found that 92 people (44%) chose internal community activities and 118 people (56%) chose external community activities. Due to the ability of the commercial communities to meet the daily sports, shopping, leisure, and other needs of older adult individuals, the older adult’s commercial activities are generally limited to their own communities. Other communities have insufficient space for community activities, shopping, and leisure. The older adult need to go to parks, squares, and shopping malls far from their communities, which results in passive isolation for the older adult with poor physical quality. Further statistics show that before retirement, 25 led cadres (12%), 103 had been workers (49%), and 82 had been freelancer workers (39%) in all communities.

### 3.2. Reliability and validity tests

**Figure 2:**
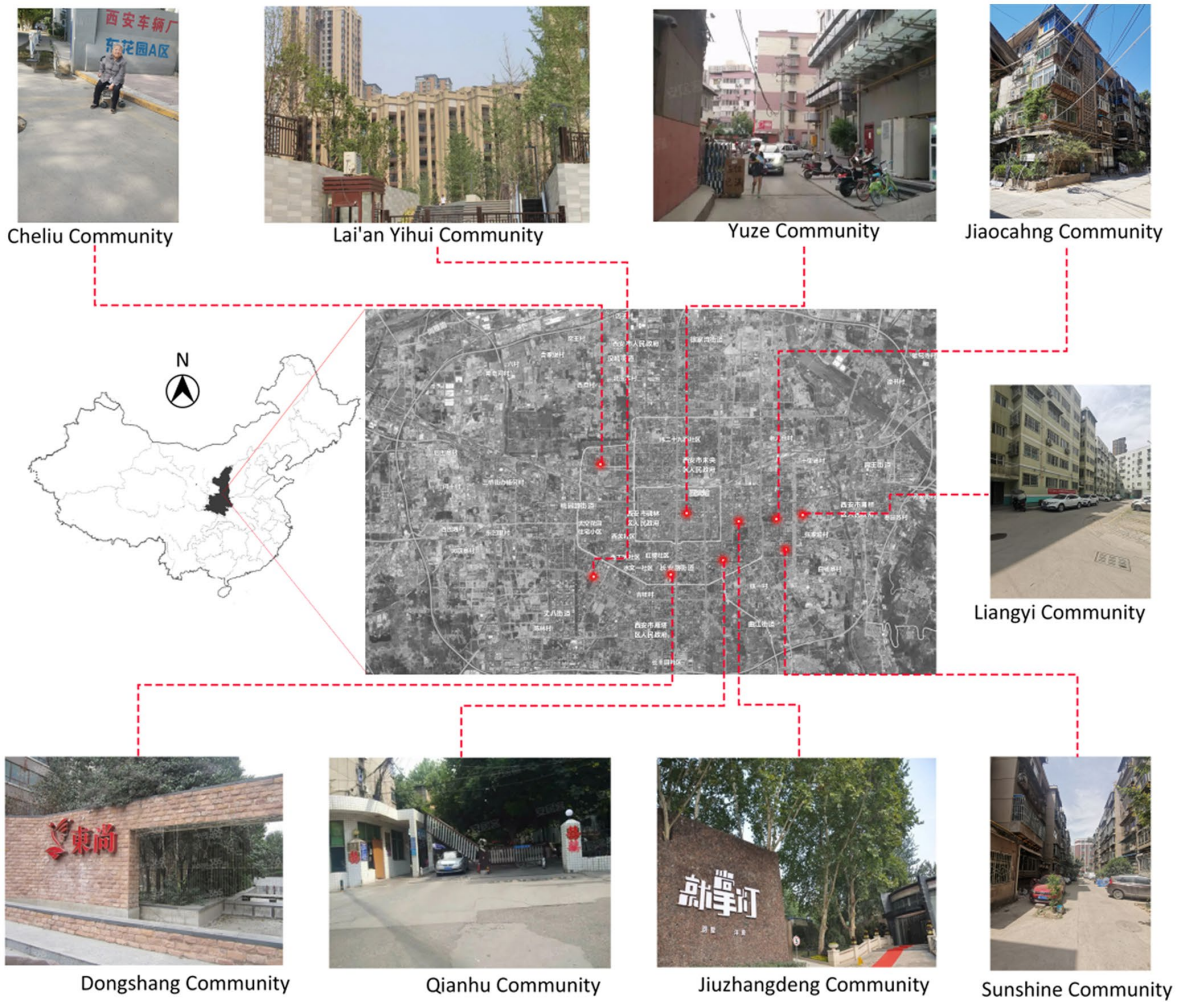
Community level.

This study primarily uses the Alpha coefficient to test reliability, and the reliability analysis is summarized in Table 2. The total alpha coefficient of the questionnaire is 0.994, and the Alpha coefficients of environmental satisfaction, environmental facilities, supporting facilities, traffic travel, and social isolation are all greater than 0.80. Thus, the sample data of this questionnaire are relatively reliable (31). The validity test is shown in Table 2. The KMO value is 0.985, and the significance of Bartlett’s sphericity test is 0.000, so the validity of the obtained data meets the standard. The combined indicators of evaluation are shown in Table 2, revealing that the data add AVE [(yi – (mxi + b)) extraction quantity] met the requirements (>0.5); the reliability CR of the combined factors met the requirement (>0.7). In summary, the scale of this study has good reliability and convergent validity. Therefore, the inventory item set is reasonable (see Figure 2).

### 3.3. Model test model fit test

**Figure 3:**
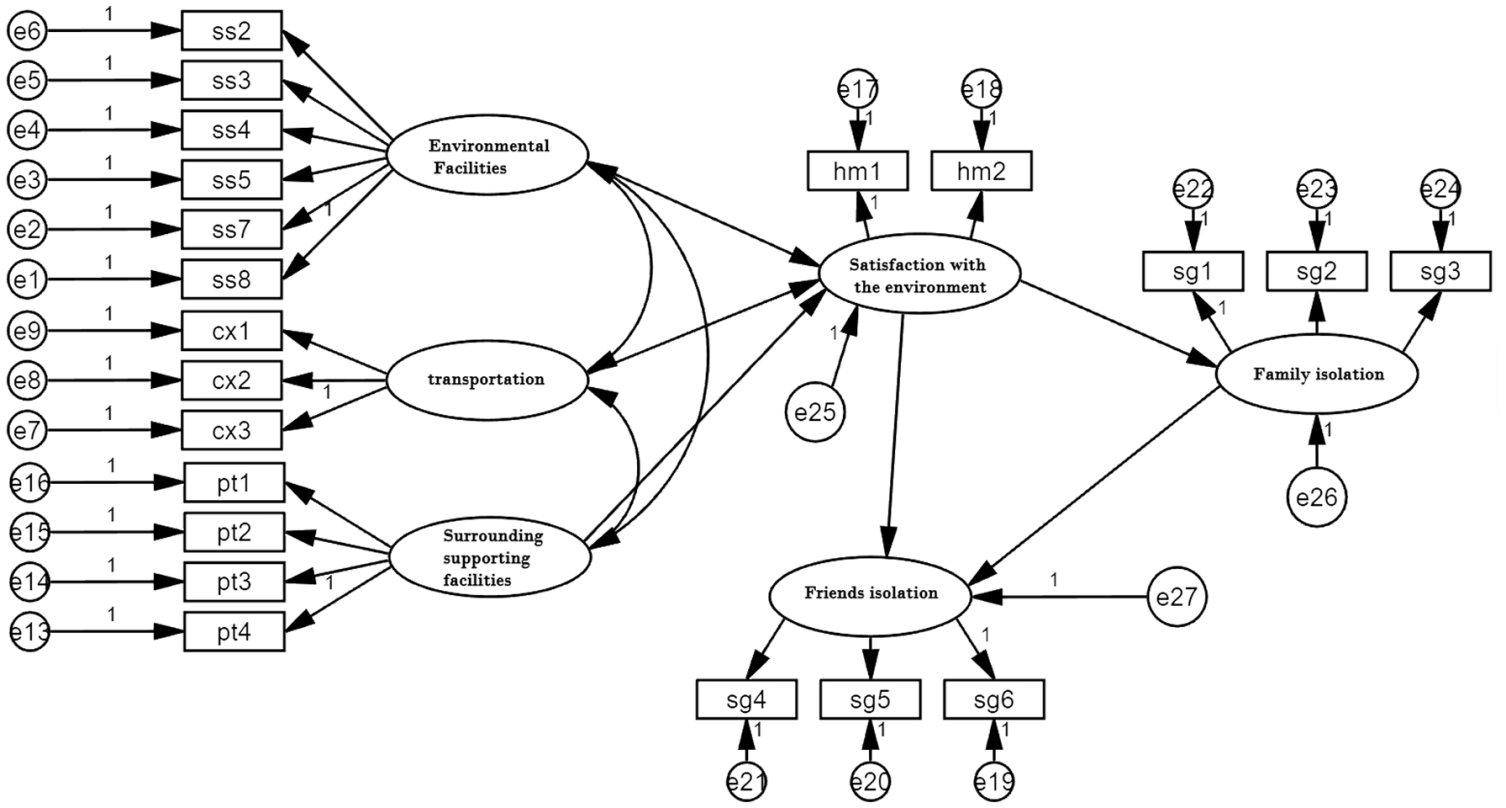
Model fitting.

In this study, Amos Graphics CLI was used to fit the model of environmental satisfaction and social isolation, as shown in Figure 3 (see Table 3).

**Table 3:** Convergent validity test.

Target	Item	Convergent validity	Component reliability	Alpha	KMO	Bartlett
		AVE	CR			
Environmental facilities	ss8	0.937	0.989	0.987	0.985	^***^
	ss7					
	ss5					
	ss4					
	ss3					
	ss2					
Transportation	cx3	0.814	0.929	0.928		
	cx2					
	cx1					
	fw2					
	fw1					
Surrounding supporting facilities	pt4	0.914	0.977	0.972		
	pt3					
	pt2					
	pt1					
Satisfaction with the environment	hm1	0.949	0.974	0.978		
	hm2					
Friend isolation	sg6	0.863	0.949	0.947		
	sg5					
	sg4					
Family isolation	sg1	0.805	0.925			
	sg2					
	sg3					

^*^P&LT, 0.05, ^**^P&LT, 0.01, ^***^P&LT, 0.001. The values not marked with ^*^ were not significant.

The specific value of the model fit test is shown in Table 4, and the chi-square/degree of freedom = 1.833 meets the requirement of a value less than 3 (32). The similarity indexes CFI and TLI and the dissimilarity indexes RMSEA and SRMR all meet the requirements of ideal values, which indicates that the model has a good fit with the data (33–35), allowing further study (see Table 5).

**Table 4:** Test of model fit.

Indicators	Model index values	Standard of indicators	Conclusion	Source of standards
CMID	423.473	The less, the better		
DF	231	The less, the better		
CMID/DF	1.833	<3 excellent <5 acceptable	Excellent	Hayduk (32)
GFI	0.849	>0.8 acceptable >0.9 excellent	Acceptable	Bagozzi and Yi (33)
AGFI	0.804	>0.8 acceptable >0.9 excellent	Acceptable	Scott (35)
CFI	0.979	>0.9 excellent	Excellent	Bagozzi and Yi (33)
TLI(NNFI)	0.975	>0.9 excellent	Excellent	
RMSEA	0.063	<0.8 excellent <0.1 acceptable	Excellent	Bagozzi and Yi (33)
SRMR	0.072	<0.08	Excellent	Hu and Bentler (34)

**Table 5:** Road test results.

Assuming that	Variable	Unstd.	S.E.	C.R.	*P*	Std. (*β*)	Results	*R*^2^
Environmental facilities	Satisfaction with the environment	0.849	0.116	7.339	^***^	0.869	Clearly, established	0.904
Surrounding supporting facilities		0.141	0.206	2.686	^*^	0.118	Set up	
Transportation		0.099	0.186	3.536	^*^	0.084	Set up	
Satisfaction with the environment	Family isolation	0.572	0.039	14.813	^***^	0.747	Clearly, established	0.718
Satisfaction with the environment	Friend isolation	0.703	0.05	14.095	^***^	0.829	Clearly, established	0.895
Family isolation		0.17	0.073	2.338	0.019	0.135	Do not set up	

^*^P&LT, 0.05, ^**^P&LT, 0.01, ^***^P&LT, 0.001. The values not marked with ^*^ were not significant.

### 3.4. Research hypothesis testing

**Figure 4:**
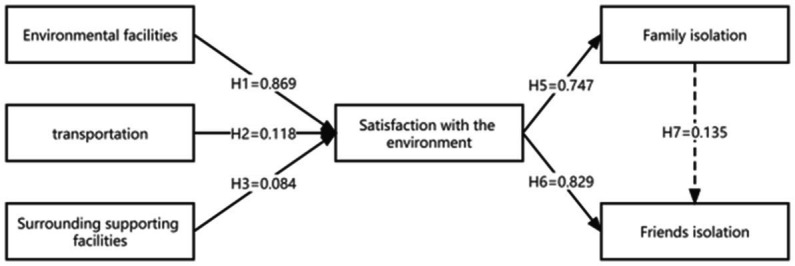
Model path coefficient output plot. Combined with Table 4, it can be concluded that community supporting facilities, traffic travel, and environmental facilities can effectively affect community environmental satisfaction. Community environmental satisfaction can also effectively affect friend isolation and family isolation in the community. However, family isolation cannot directly affect friend isolation, and there is no significant causal relationship between them.

The model was tested to determine the relationship between environmental satisfaction and social isolation. The results of the data analysis show that the *R*^2^ of the model with environmental satisfaction as the dependent variable is 0.904, the *R*^2^ of the model with family isolation as the dependent variable is 0.718, and the *R*^2^ of the model with friend isolation as the dependent variable is 0.895. The above indicators meet the standard requirements. This research model has a certain predictive effect on the relationship between environmental satisfaction and social isolation. The final path coefficients and significance level results of this study are shown in Figure 4. The solid line indicates that the hypothesis is true, and the dashed line indicates that the hypothesis is not true.

## 4. Discussion

### 4.1. Impact analysis of community environmental satisfaction

Community environmental facilities (*β* = 0.869, *p* < 0.001), transportation (*β* = 0.084, *p* < 0.005), and community supporting facilities (*β* = 0.118, *p* < 0.005) positively and directly affected environmental satisfaction and together explained 90.4% of the variance in environmental satisfaction (H1, H2, and H3 were confirmed). That is, community environmental facilities, community supporting facilities, and transportation play an important role in the community environmental satisfaction of the older adult.

Environmental facilities scored highly in the community environment satisfaction of the older adult because the older adult are more inclined to walk and enjoy leisure in their community during their daily activities (36). Good traffic continuity and road comfort are conducive to promoting older adult walking and leisure (37), and cleanliness, an active population, and the environment are important factors affecting walking (38). The community environment and facilities can affect the quality of life of the older adult (39). Community facilities are important because older adult people like to walk for entertainment and travel. Therefore, transportation is more concerned with whether department stores, supermarkets, hospitals, and other supporting areas are within walking distance. Transportation scored lowest in community environment satisfaction. This is because most older adult people are not willing to drive or take public transportation, and hitchhiking is the most popular mode of transportation for older adult people (40).

In conclusion, community environmental facilities play a dominant role in the evaluation of the environmental satisfaction of the older adult in the community. Therefore, in future aging designs, priority should be given to the internal green environment, hard decoration, and facilities.

### 4.2. Associations between environmental satisfaction and social isolation

Environmental satisfaction directly affected friend isolation (*β* = 0.829, *p* < 0.001) and family isolation (*β* = 0.747, *p* < 0.001). Therefore, the more satisfied the older adult are with the environment, the less social isolation occurs (H4 and H5 are confirmed). Moreover, environmental satisfaction had a greater effect on friend isolation and a slightly smaller effect on family isolation. Among them, although the environment of some industrial communities is poor, most of the community members are factory workers who are familiar with each other. Therefore, family isolation is more significant, but friend isolation is not obvious. There was no social isolation among the cadre leaders. This is because cadre leaders are generally highly educated and better at communicating with their children, neighbors, and friends, so their social network is strong.

Studies have found that most older adult people in China participate in square dance community entertainment activities, which are in high demand (41); through these social activities, they can effectively inhibit social segregation and improve their happiness index (42). However, scholars have also found that the current urban communities leave nearly half of the older adult individuals isolated (43).

Therefore, to prevent the social isolation of older adult individuals, we should first improve the green landscape environment of the community. Second, to meet the needs of older adult community recreational activities, corresponding facilities should be added. Finally, the continuity of community road traffic and road comfort should be optimized to encourage older adult people to travel outdoors.

### 4.3. Limitations of the study

There are still some shortcomings in this study. First, all participants in this study were older than 60 years old. Due to the great changes in social networks after retirement, the older adult become a high-incidence group for social isolation. Older adult individuals are the most frequent users of community public spaces. However, other age groups may also face social isolation, so to fully explore the relationship between community environmental satisfaction and social isolation, we should consider further related research on other age groups. Second, due to the limitations of the structural equation model, this study studied only the Xi’an area. However, this research method is applicable to any region. Therefore, this research method should be used for future research and analysis in other regions. Finally, this study primarily focuses on the older adult in urban communities. Since the older adult in rural areas of China primarily live in self-built houses, their lifestyles, life behaviors, and social interactions are quite different from those of the older adult in urban communities of China, so a comparative analysis cannot be carried out. Therefore, the follow-up study can focus on the living habits and environment of the older adult in rural areas of China.

## 5. Conclusions and suggestions

Starting from the community environment, this study is the first to comprehensively construct a structural equation model of the impact of community environmental satisfaction on the social isolation of older adult individuals.

First, there are significant differences in the focus on the older adult’s environmental satisfaction. Environmental facilities have a much higher impact on environmental satisfaction than transportation and supporting facilities, while transportation has the least impact on environmental satisfaction. Second, the relationship between the community environment and the social isolation of the older adult was revealed; that is, environmental satisfaction had a direct positive effect on social isolation, and the effect of environmental satisfaction on friend isolation was more obvious. The results showed that the older adult paid more attention to the environmental facilities of the community, such as greening, sanitation, equipment, and road paving. Most older adult people do not need to go far away, so they do not care much about the convenience of transportation. Older adult people prefer to have complete facilities around the community, including hospitals, supermarkets, and shopping malls. There is no direct effect of family isolation or friend isolation on older adult individuals, but a clean, comfortable, and well-equipped community can effectively decrease social isolation. This study provides a new idea for the prevention of social isolation and provides data support for the design of aging environments in the future.

Based on the results of this study, three suggestions are proposed for the prevention of social isolation and age-appropriate design. A high-quality community environment and landscape should be provided, diversified activity facilities should be improved, road continuity and comfort should be ensured, and outdoor activities and communication should be promoted for the older adult in the community.

## Data availability statement

The raw data supporting the conclusions of this article will be made available by the authors, without undue reservation.

## Author contributions

SM, GL, and YD: conceptualization and writing—review and editing. SM: methodology, software, formal analysis, investigation, data curation, and writing—original draft preparation. SM and GL: resources. All authors contributed to the article and approved the submitted version.

## Conflict of interest

The authors declare that the research was conducted in the absence of any commercial or financial relationships that could be construed as a potential conflict of interest.

## Publisher’s note

All claims expressed in this article are solely those of the authors and do not necessarily represent those of their affiliated organizations, or those of the publisher, the editors and the reviewers. Any product that may be evaluated in this article, or claim that may be made by its manufacturer, is not guaranteed or endorsed by the publisher.
